# Identification of Survival and Therapeutic Response-Related Ferroptosis Regulators in Bladder Cancer through Data Mining and Experimental Validation

**DOI:** 10.3390/cancers13236069

**Published:** 2021-12-02

**Authors:** Pu Zhang, Zijian Liu, Decai Wang, Yunxue Li, Yuan Zhang, Yajun Xiao

**Affiliations:** 1 Department of Urology Surgery, Union Hospital, Tongji Medical College, Huazhong University of Science and Technology, 1277 Jiefang Avenue, Wuhan 430022, China; d202081801@hust.edu.cn (P.Z.); jmlprocks@163.com (Y.L.); 2 Cancer Center and State Key Laboratory of Biotherapy, Department of Head and Neck Oncology and Department of Radiation Oncology, West China Hospital, Sichuan University, Chengdu 610041, China; 2020324025312@stu.scu.edu.cn; 3 Department of Emergency Surgery, Union Hospital, Tongji Medical College, Huazhong University of Science and Technology, 1277 Jiefang Avenue, Wuhan 430022, China; decaiwang_2020@163.com; 4 Clinical Research Center of Kidney Disease in Sichuan Province, Department of Nephrology, Sichuan Provincial People’s Hospital, Medicine of School, University of Electronic Science and Technology of China, Chengdu 611731, China

**Keywords:** ferroptosis, tumor environment, therapeutic efficacy, prognosis, bladder cancer

## Abstract

**Simple Summary:**

Based on machine learning methods, we constructed a prognostic signature to calculate the survival probability of BLCA and to further investigate the underlying mechanism of the ferroptosis-related signature. We found that the signature was not only correlated with the prognostic value but was also associated with the tumor microenvironment (TME), tumor mutation burden (TMB) and the curative outcomes of both immunotherapy and chemotherapy. Furthermore, we proved the reliability of the signature in some external datasets and built a risk score evaluation nomogram for clinical use.

**Abstract:**

Ferroptosis has been reported to regulate tumorigenesis, metastasis, drug resistance and the immune response. However, the potential roles of ferroptosis regulators in the advancement of bladder cancer remain to be explored. We systematically evaluated the multidimensional alteration landscape of ferroptosis regulators in bladder cancer and checked if their expression correlated with the ferroptosis index. We used least absolute shrinkage and selection operator regression to form a signature consisting of seven ferroptosis regulator. We confirmed the signature’s prognostic and predictive accuracy with five independent datasets. A nomogram was built to predict the overall survival and risk of death of patients. The relative expression of the genes involved in the signature was also clarified by real-time quantitative PCR. We found the risk score was related to tumor progression and antitumor immunity-related pathways. Moreover, there existed negative association between the relative antitumor immune cell infiltration level and the risk score, and higher tumor mutation burden was found in the group of lower risk score. We used The Tumor Immune Dysfunction and Exclusion database and IMvigor210 cohort having immunotherapy efficacy results to confirm the prediction function of the risk score. Furthermore, the ferroptosis regulator signature could also reflect the chemotherapy sensitivity of bladder cancer.

## 1. Introduction

Bladder cancer (BLCA), an aggressive and highly recurrent tumor in the urinary system, causes high morbidity and mortality rates [1]. According to the 2015 China cancer statistics, there were approximately 80,500 new cases and 32,900 deaths from BLCA in the past few years [2]. Based on histological differentiation and whether the tumor cells are found in the muscle layer, we can divide bladder tumor into low- and high-grade or nonmuscle-invasive and muscle-invasive subgroups, respectively, which have different prognoses and metastatic risks [3,4]. Although well-established operative treatment and chemotherapy options have allowed many cases of BLCA to have a good prognosis, the recurrence and metastasis of BLCA are major causes of treatment failure. Therefore, the specific mechanisms leading to the progression and metastasis of BLCA still need deeper exploration and research to offer reliable theoretical evidence for healing this type of cancer near future.

It is widely reported that programmed cell death, like apoptosis and autophagy, owns a crucial role in tumorigenesis, metastasis and oncotherapy [5,6,7]. The role of apoptosis or autophagy in BLCA has been well elucidated, and therapeutic methods based on inducing this way of cell death, for example cisplatin and gemcitabine, have achieved tremendous clinical benefits [8,9]. Ferroptosis featured by the stock of lipid peroxidation, is different from traditional apoptosis, autophagy or necrosis [9,10]. In recent years, induction of cancer cell death through triggering ferroptosis might be an alternative treatment to malignancies resistant to traditional therapies [11,12]. In particular, the role of ferroptosis in cancer immunotherapy has been gradually discovered and thoroughly investigated [13]. 

Integrative analysis of ferroptosis regulators will enhance our comprehension of the function and value of ferroptosis in the aspects of progression, metastasis of tumor and treatment approaches. Previous studies have constructed ferroptosis-associated gene signatures to predict overall survival and explore biological function in multiple tumors, including glioma and hepatocellular carcinoma [14,15,16]. But the molecular function and clinical implications of ferroptosis in BLCA remain uncertain, and further study is essential to explore the function of ferroptosis in BLCA to offer possibility for manufacturing new antitumor drugs. 

## 2. Methods

### 2.1. Data Collecting and Processing

We extracted data associated with BLCA from public databases such as the Gene-Expression Omnibus (GEO) and The Cancer Genome Atlas (TCGA). In total, 4 GEO datasets (GSE13507, GSE32548, GSE32894, GSE48075), TCGA-BLCA and the IMvigor210 cohort were used in this study. For the microarray data, we downloaded the normalized matrix files and clinical phenotype from the GEO database. For the data from TCGA, the clinical phenotype and copy number variation (CNV) data was retrieved from the XENA database. The R package TCGAbiolinks was used for somatic mutation data. We obtained gene transcriptional and clinical information of 348 patients experiencing metastatic urothelial cancer who accepted immune checkpoint inhibitor treatment from the database http://researchpub.gene.com/IMvigor210CoreBiologies. (accessed on 2 February 2021) A total of 113 ferroptosis regulators were extracted from an online website, FerrDb (2 February 2021) including 49 suppressors, 61 drivers and 3 markers with validated confidence levels in the *Homo sapiens* experiment, and the specific information of these genes is shown in Table S1. 

### 2.2. Construction of the Ferroptosis Regulators Signature in BLCA

With the expression profiles of ferroptosis regulators, LASSO regression method was implemented so that we could recognize the most representative ferroptosis regulators owning prognostic value in the training dataset. Subsequently, a prognostic signature was built on the basis of the candidate regulators generated from the filtration process mentioned above. The risk score was calculated using the following equation: Risk Score = (Coef_i_ × Exp_i_). The accuracy and efficiency of the signature was estimated through the receiver operating characteristic (ROC) curve in a time-dependent way.

### 2.3. Construction of a Nomogram on the Basis of the Risk Scores and Clinical Parameters

Univariate and multivariate Cox regression analysis was implemented so that we could choose survival-related parameters in combination of risk scores and clinical parameters. Then, a nomogram made up of the risk score and clinical parameters was built through the R package “rms” to predict the survival probability and death odds. Through a calibration plot, we tested the predictive accuracy. 

### 2.4. Calculation of TME Cell Infiltration Abundance

The CIBERSORT algorithm https://cibersort.stanford.edu/ (accessed on 2 February 2021) utilized to estimate the infiltration levels of 22 types of immune cells in BLCA [17]. Furthermore, we performed a single sample gene set enrichment analysis (ssGSEA) algorithm to calculate the relative abundance of immune cells in the samples of bladder tumor. In addition, we present the sets of immune cell markers (Table S2) [18].

### 2.5. Prediction of Possible Immunotherapy Outcomes for Patients

On the basis of pretreatment gene expression in tumor samples, the Tumor Immune Dysfunction and Exclusion (TIDE) database could forecast the possible immunotherapy outcomes of patients [19]. The TIDE value was calculated and utilized to evaluate the possible results of an immunotherapy, and the cut-off of this value was defaulted as 0.

### 2.6. Chemotherapeutic Response Prediction 

The chemotherapeutic response for each patient was predicted on the basis of the Genomics of Drug Sensitivity in Cancer (GDSC), https://www.cancerrxgene.org/ (accessed on 2 February 2021). We implemented the prediction process by utilizing the R package “pRRophetic” [20].

### 2.7. Calculation of the Ferroptosis Index

The ferroptosis index (FPI) to indicate the ferroptosis level was established according to the expression data for genes of the ferroptosis core machine. We calculated the enrichment score (ES) of the gene set that positively or negatively regulated ferroptosis using ssGSEA, and we utilized the ferroptosis index (FPI) calculated as follows: FPI = ES (positive)–ES (negative) [21] to computationally dissect the ferroptosis levels/trends of the tissue samples. 

### 2.8. The Real-Time Quantitative PCR Analysis 

Human normal bladder tissues and human bladder tumor tissues were collected from the patients experiencing radical cystectomy in Wuhan Union Hospital. The normal tissues were obtained after we performed radical cystectomy in patients with bladder cancer. We open the bladder and take down the tissues next to the cancer tissues. In addition, these tissues were placed in liquid nitrogen immediately. The criteria was that the normal tissues were 5 cm far from the cancer tissues and the tumor cells were not found invading in the normal tissues in the pathological examination. Our department of pathology performed immunohistochemistry staining and found GATA-3(+), CK7(+), CK20(+), P40(+) in the tumor tissues. We extracted the total RNA from 12 pairs of frozen bladder specimen through TRIZol reagent (Invitrogen, 15596026) following an instruction provided by the manufacturer Thermo Fisher Sicentific, Wuhan, China. The SYBR Green One-Step qRT-PCR kit (Invitrogen, 11736059) was utilized to measure total RNA (100 ng). The specific details of primers were shown in Table S3. The relative expression of these genes in normal and tumor tissues were presented in “PCRdata”, and the clinicopathological data of the 12 pairs of tissues was presented in “Clinical pathological data for the tissues used for PCR”.

### 2.9. Statistical Analysis

Spearman correlation analysis was put into effect to estimate the correlation coefficient between two indicators in the study. The Wilcoxon test was performed to compare the variation between two different groups. We performed univariate Cox regression analysis to evaluate the prognostic value of ferroptosis regulators. We utilized the “survminer” package to divide the risk score and the expression value of the ferroptosis related genes. What is more, we repeatedly tested all potential cutting points to search for the maximum rank statistic. In addition, we assigned the patients into two groups on the basis of the maximum selected log-rank statistics. We conducted GSVA enrichment analysis utilizing the “GSVA” R package [22] on the basis of hallmark gene sets obtained from the database named MSigDB to assess the enrichment score of the curated pathways. Spearman correlation analyses were employed to calculate correlation coefficients. All statistical analyses were two-sided. *p* < 0.05 was believed to be that the difference is statistically significant.

## 3. Results

### 3.1. Multidimensional Alteration Landscape of Ferroptosis Regulators in BLCA

**Figure 1:**
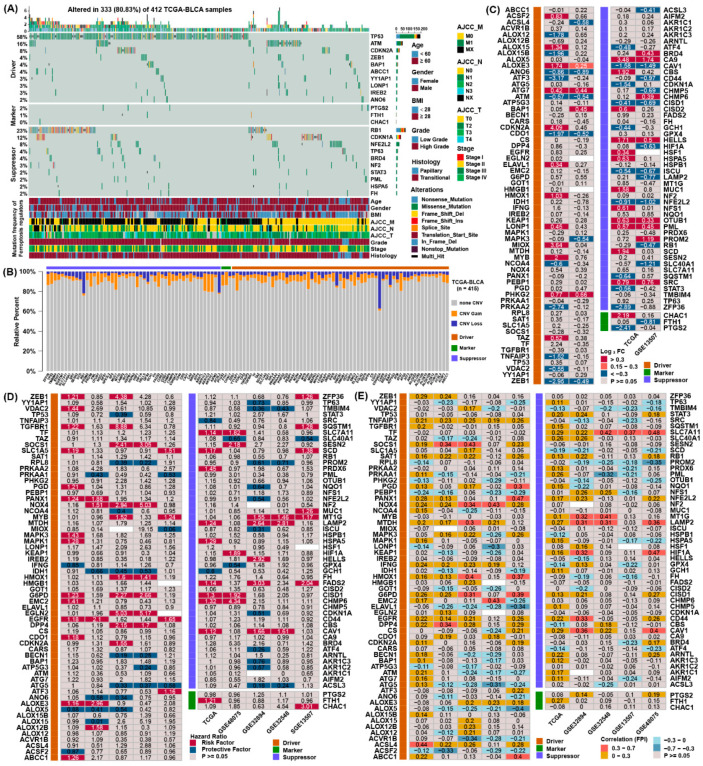
Multidimensional alteration landscape of ferroptosis regulators in BLCA. (**A**) The ferroptosis regulators with mutations were shown in BLCA patients from TCGA. The mutation types and basic information of patients were also annotated in the right of figure. (**B**) Bar graphs indicating the relative percentage of CNV gain, loss and the non-CNV of ferroptosis regulators in the TCGA. The orange bars represented genes with CNV gain, blue bars represented CNV loss and gray bars represented no changes in CNV. (**C**) Differential expression analysis of 113 ferroptosis regulators between normal and BLCA tissues. The number in the column represents the log_2_ fold change, red represents higher expression in cancer, and blue represents lower expression in cancer. (**D**) The prognostic analyses for ferroptosis regulators in five independent datasets and hazard ratios are shown in the column. The red columns represented risk factors, while blue columns represented protective factors. (**E**) Correlation analysis between ferroptosis regulators, the ferroptosis index, and the Spearman correlation coefficients are shown in the column.

To obtain the genetic alterations of ferroptosis regulators in BLCA, the prevalence of nonsilent somatic mutations was assessed in TCGA. Among the 412 BLCA samples in TCGA, 333 (80.83%) had mutations in ferroptosis regulators (Figure 1A). Among them, the mutation frequencies of TP53 and ATM were the highest for the ferroptosis driver genes, and RB1 and CDKN1A were the highest for the ferroptosis suppressor genes. However, the mutation frequency of marker genes was relatively low in bladder cancer. Moreover, we examined copy number alterations of these regulators and found AKR1C1/2/3, MUC1 and YY1AP1 owned a lot of copy number variation (CNV) gain, while CDKN2A, RB1 and ACSL3 owned the highest frequency of CNV loss (Figure 1B). 

To confirm whether the expression of ferroptosis regulators were influenced by these genetic variations, we contrasted the gene expression pattern between normal tissues and BLCA samples in two datasets (Figure 1D). Compared to normal bladder tissue, regulators with CNV gain, such as ATG7, PHKG2, CA9, OTUB1 and PML, were markedly highly expressed in BLCA tissues, while regulators with CNV loss, such as ATM, CISD1 and CDO1, were markedly lower in BLCA tissues. However, the expression of some regulators was not consistent with the change in CNV, demonstrating CNV was not the independent element to determine their expression. 

To evaluate the prognostic value of ferroptosis regulators, we assessed their hazard ratios in five independent datasets (Figure 1D). Many prognostic factors were verified in the five independent datasets, indicating that the expression of ferroptosis regulators might be related to the prognosis of patients with BLCA. To further ascertain the association between these regulators and ferroptosis, we employed FPI to conduct correlation analyses with their expression (Figure 1E). The results showed that most regulators displayed a consistent relationship with FPI, indicating that the expression of these regulators might be associated with the level of ferroptosis. These analyses demonstrated the discrepancy in the expression of ferroptosis regulators may own an important role in the prognosis and advancement of BLCA.

### 3.2. Constructing a Ferroptosis Regulators Signature of BLCA

**Figure 2:**
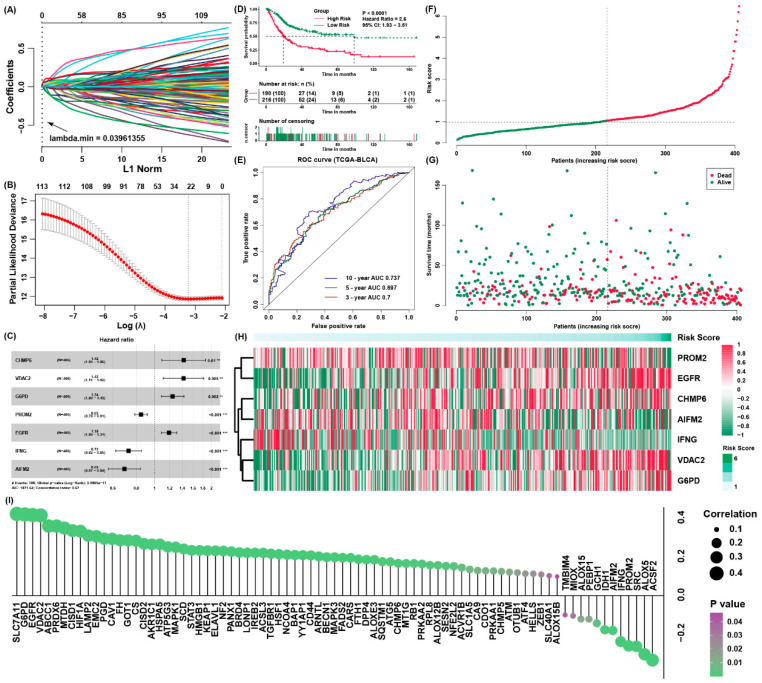
Construction of a ferroptosis regulator signature of BLCA. (**A**,**B**) The LASSO model and the LASSO coefficient profiles are presented; (**C**) independently prognostic parameters of the ferroptosis regulator signature; (**D**) the survival curves of the low-risk and high-risk group; (**E**) the ROC curves for overall outcomes of three, five and ten years; (**F**) the risk score distribution in the patients with BLCA from TCGA; (**G**) the vital status of patients from different group of risk scores; (**H**) the heatmap for the expression of genes in the signature; (**I**) the possible correlation between the expression of ferroptosis regulators and the risk score.

**Figure 3:**
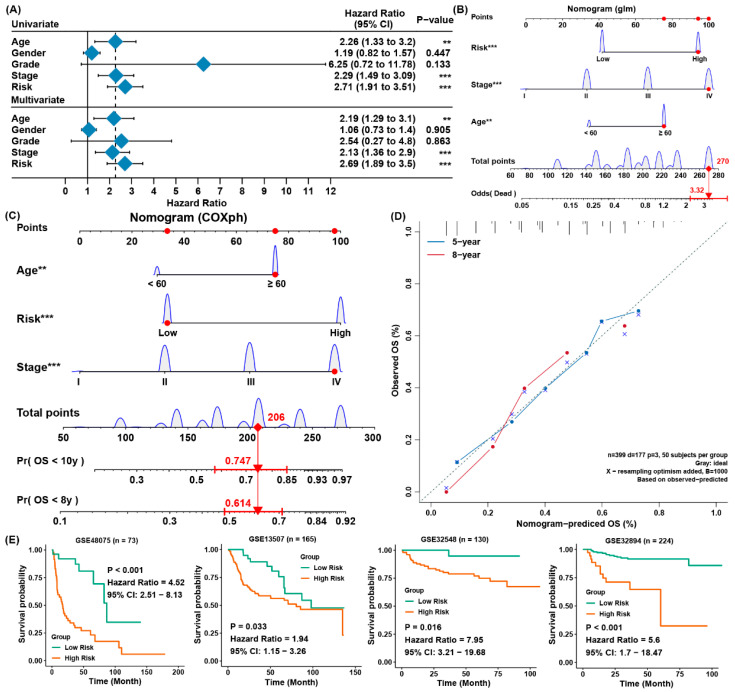
Establishing a predictive nomogram and external verification. (**A**) Univariate and multivariate Cox analysis of clinical parameters and risk scores with outcome of overall survival of the patients with BLCA from TCGA; (**B**) the nomogram to predict the rates of death of patients with BLCA; (**C**) the nomogram to predict overall survival outcomes of BLCA patients for five and ten years; (**D**) the calibration curve to evaluate the accuracy of the nomogram constructed based on gene signature; (**E**) verification of the ferroptosis-associated gene signature in four external cohorts from GEO. ** *p*< 0.01, *** *p* < 0.001.

We applied the Lasso algorithm to recognize a set of 23 candidate prognostic ferroptosis regulators (Figure 2A,B), and after that we implemented multivariate Cox regression analysis to construct a prognostic signature for the training cohort (Figure 2C, Table S4). Moreover, we calculated the risk score of each patient. On the basis of the best cut-off point of the risk scores, we assigned all samples to high-risk and low-risk groups separately. The patients owning low-risk scores have better survival results than the patients from higher risk score group (Figure 2D), The AUC of the signature was 0.7, 0.697 and 0.737 for survival outcomes of 3, 5 and 10 years, respectively (Figure 2E). Additionally, cancer-associated death rose and the number of surviving patients fell with risk score enhanced. In addition, we use heatmap to present the expression value of every candidate gene in the formula which is associated with the risk score (Figure 3F–H). We also evaluated the relationship between the risk score and the expression of all ferroptosis regulators (Figure 3I), and we discovered other regulators, such as SLC7A11, were also potential regulators associated with the risk score.

### 3.3. The Establishment of a Predictive Nomogram and External Testification

For the sake of forecasting the survival possibility of patients with BLCA, we constructed a nomogram considering clinicopathological covariates. Through the univariate and multivariate Cox regression analysis (Figure 3A), the nomogram predicted the death odds of patients (Figure 3B) and predicted the overall survival rates for five and 10 years (Figure 3C). In contrast to the ideal model, the calibration plot predicting OS outcomes for five and 10 years have relatively good results (Figure 3D). Moreover, the four external verification groups GSE32894 GSE48075, GSE13507, and GSE32548 were also employed for the same analysis, and the risk scores were also computed according to the same signature. Compared to the patients from low-risk group, patients from the high-risk group owned a greatly lower OS rate (Figure 3E). This was in accordance with the those results of the training set, indicating the ferroptosis-associated gene signature was able to precisely forecast the survival of patients with BLCA.

### 3.4. Functional Characteristics of the Ferroptosis Regulator Signature

**Figure 4:**
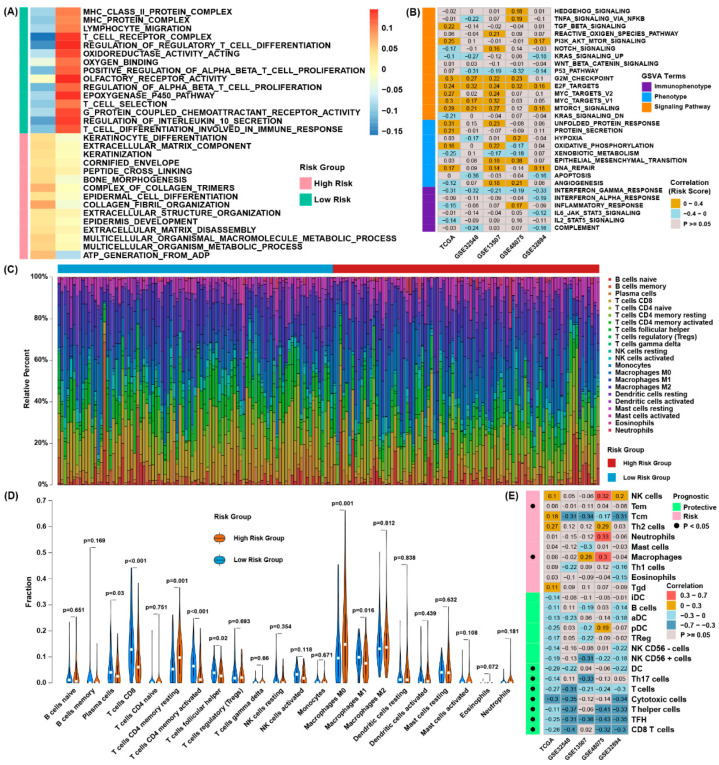
Functional characteristics of the ferroptosis regulator signature. (**A**) The result of GSEA showed different enriched gene sets between low-risk and high -risk group; (**B**) the correlation coefficient between the scores of GSVA and the risk scores in five datasets; (**C**) the relative infiltration percentage of 22 immune cells of each patient from TCGA through CIBERSORT; (**D**) the violin plot showing the relative infiltration level of immune cells based on the low-risk and high-risk group; (**E**) correlation analysis between the abundance of the immune cell enrichment score and the risk score in five independent datasets.

We implemented GSEA in the TCGA cohort to investigate the possible mechanism of the signature, and we found that low-risk group was significantly related to T cell- and MHC-associated pathways, and the group with the higher risk score was importantly related to extracellular matrix component-related pathways (Figure 4A). To verify the related pathways between risk groups, we conducted GSVA in four verification datasets and the TCGA dataset to evaluate the alterations of the pathways (Figure 4B). The risk score was related to E2F, MYC targets and G2/M checkpoints, which were highly relative to tumor progression and metastasis, and conversely related to apoptosis and interferon-γ response pathways. Then, utilizing CIBERSORT algorithm we calculated the immune infiltration levels of immune cells in BLCA between the high-risk and low-risk groups (Figure 4C). The outcomes revealed that CD8 T cells, activated memory CD4 T cells and M1 macrophages were tremendously gathered in the group of lower risk score (Figure 4D). Correlation analysis evaluating the association between the risk score and immune cell enrichment score revealed the relative infiltration levels of CD8 T cells, follicular helper T (TFH) cells, T cells and cytotoxic cells, when assessed using the ssGSEA approach, were highly negatively related to the risk score (Figure 4E). The above results indicated the unfavorable survival outcomes of the high-risk group might be relative to tumor progression and the favorable survival outcomes of the group of lower risk scores may be associated with higher infiltration levels of CD8 T cell and other antitumor immune cells.

### 3.5. Evaluation Efficacy for Immunotherapy of Ferroptosis Regulator Signatures

**Figure 5:**
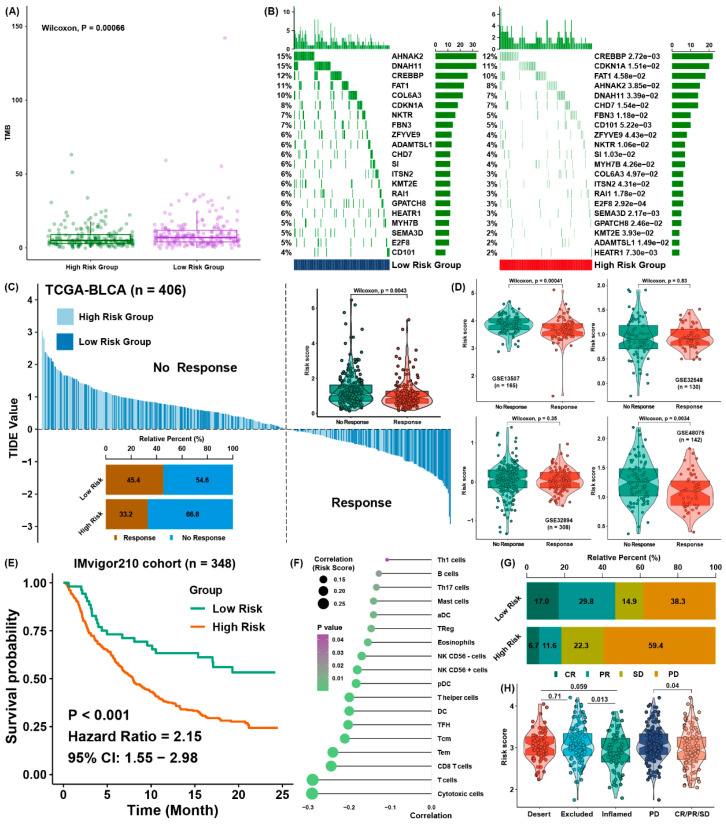
Evaluation efficacy for immunotherapy of the ferroptosis regulator signature. (**A**) The different levels of TMB between low-risk group and high-risk groups; (**B**) the mutation frequency of different risk scores groups; (**C**) the TIDE value of each sample with BLCA is presented based on distinct risk score groups, and the distribution of efficacy is shown on the bottom left. The levels of the risk score in different response groups is also presented; (**D**) the abundance of risk scores in groups with distinct response results in four external validation datasets is shown; (**E**) survival analysis of the risk score in the IMvigor210 cohort; (**F**) correlation analysis of the risk score and relative immune cell infiltration level in the IMvigor210 cohort; (**G**) relative percentages of CR, PR, SD and PD patients in different risk groups; (**H**) the abundance of risk scores in different groups in the IMvigor210 cohort.

According to the report, patients owning a relatively higher tumor mutation burden (TMB) might gain profit from immunotherapy possibly because of more neoantigens [23]. After processing the mutation files in TCGA dataset of BLCA, we found the low-risk group possessed a higher TMB in contrast to the group of higher risk scores (Figure 5A), which indicated the group of lower risk scores may gain profit if they receive immunotherapy. After that, the distribution differences of somatic mutations were analyzed between the two groups. The mutation rates were higher in the low-risk group (Figure 5B). 

To predict the immune response, the patients in the cohort of TCGA were assigned into two groups by a response or no-response prediction for immunotherapy. We found the response group had a lower risk score and that the relative percentage of response samples was larger in the group of lower risk score (Figure 5C). To verify the prediction efficacy, we discovered the risk score was greatly lower in the low-risk group in GSE13507 and GSE48075 (Figure 5D). 

Lastly, our study estimated the prediction effect of the ferroptosis signature in the IMvigor210 cohort, which received immunotherapy treatment. As expected, the risk score was a risk factor (Figure 5E) and was conversely related to the relative infiltration level of cytotoxic T and CD8 T cells (Figure 5F). The percentage of CR and PR patients was higher in the low-risk group (Figure 5G), and the risk score was lower in the inflamed phenotype and CR/PR/SD groups (Figure 5H).

### 3.6. Possible Therapeutic Value of the Ferroptosis Regulator Signature

**Figure 6:**
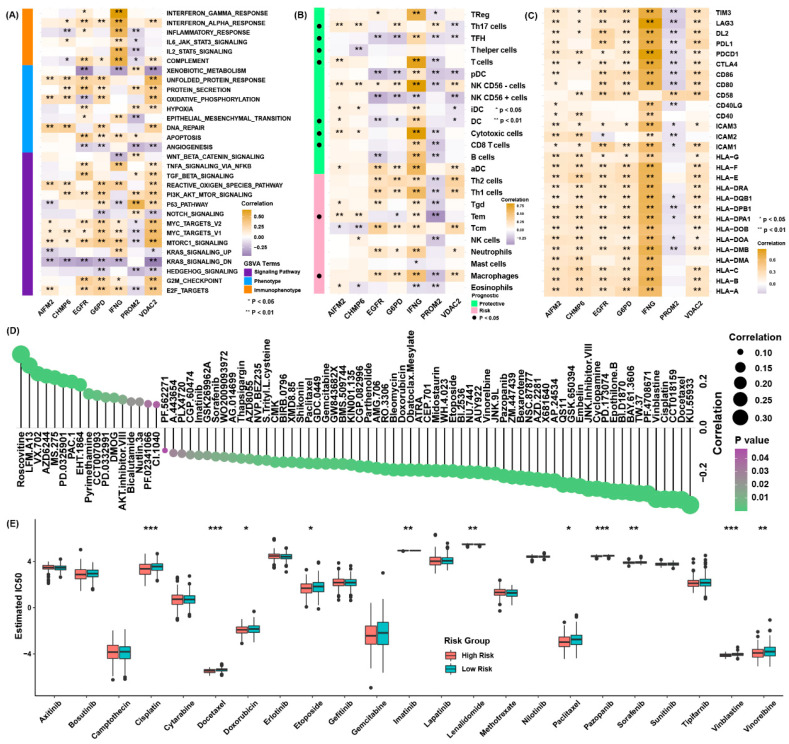
Potential therapeutic value of the ferroptosis regulator signature. (**A**) The correlation coefficients between the scores of the GSVA and the expression of members in the ferroptosis regulator signature in the TCGA cohort; (**B**) correlation analysis between the abundance of immune cell enrichment scores and the expression of members in the ferroptosis regulator signature in the TCGA cohort. The dots in the left columns represented the prognostic values of immune cell infiltration levels with statistic significances; (**C**) correlation analysis between the expression of MHC molecules, costimulatory factors, adhesion factors and the expression of members of the ferroptosis regulator signature in the TCGA cohort; (**D**) correlation analysis between the estimated IC50 value of 138 drugs in the GDSC cohort and the risk score in the TCGA cohort; (**E**) the estimated values of IC50 of some frequently adopted drugs for chemotherapy are presented in the distinct risk scores groups. * *p*< 0.05, ** *p* < 0.01, *** *p* < 0.001.

To deeply explore the function of the risk score system, the genes involved in the signature were subjected to functional analysis. Correlation analysis showed that all of the members were associated with pathways correlated with the risk score, such as E2F, MYC targets, G2/M checkpoint and immune-related pathways (Figure 6A). For immune cell infiltration, IFNG was actively related to the infiltration levels of cytotoxic and CD8 T cells, and PROM2 was the opposite (Figure 6B). A previous study showed that DCs play a role in presenting antigen and activating naive T cells. The enhanced expression of MHC molecules, adhesion factors and costimulatory factors also cause activation of native T cells [24]. The expression of MHC molecules, adhesion molecules and costimulatory molecules were positively related to the expression of members of the ferroptosis signature, except for PROM2 (Figure 6C). 

To explore the influence of the risk score on the drug response, the estimated IC50 value of 138 drugs in the GDSC database were calculated in the TCGA-BLCA cohort (Figure 6D). We discovered most drugs were more sensitive in the group of higher risk scores, and we sought out the chemotherapy and targeted drugs that were frequently used clinically (Figure 6E). We found the group of higher risk score might be more susceptible to cisplatin, docetaxel, doxorubicin, etoposide, vinblastine, etc. Together, these results imply that the ferroptosis signature is related to drug sensitivity. Therefore, the ferroptosis regulator risk score might be a possible indicator for choosing appropriate treatment methods.

### 3.7. The Experimental Validation of the Expression of the Genes of the Signature

**Figure 7:**
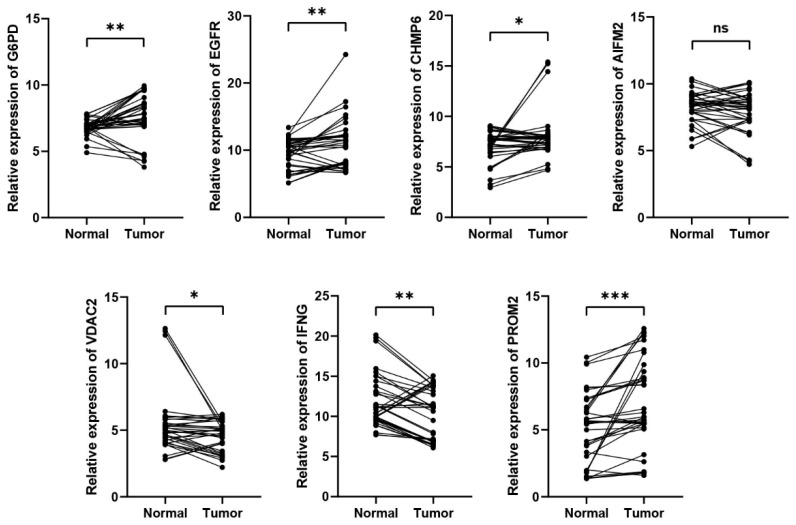
Experimental verification of the expression levels of the genes of the signature between normal bladder tissues and bladder cancer tissues through qRT-PCR. * *p* < 0.05, ** *p* < 0.01, *** *p* < 0.001.

To better validate the results of bioinformatic analysis. We collected twelve paired normal bladder tissues and bladder cancer tissues (Figure 7). The results were presented in Figure 7. It demonstrated that the expression of G6PD, EGFR, CHMP6 and PROM2 were elevated in the tumor tissues. In addition, the expression of VDAC2 and IFNG were decreased in the tumor tissues. Moreover, there is no significant difference in the expression of AIFM2 between normal bladder tissues and bladder cancer tissues. 

## 4. Discussion

We conducted this research to construct a scoring system based on ferroptosis regulators and investigate the underlying biological mechanisms including pathways variation and tumor microenvironment alteration within the signature through data mining and experimental research. In this study, to fully understand the significant role of ferroptosis in BLCA, detailed ferroptosis regulatory genes were collected and filtered from a well-known ferroptosis database FerrDb zhounan.org (2 February 2021). The genetic and expression variation landscape of ferroptosis regulators demonstrated a high heterogeneity of ferroptosis regulators with various genetic modification patterns and prognostic value in BLCA, indicating that the differentially expression of ferroptosis regulators has potential roles in the initiation and progression of BLCA.

Some articles also construct a signature only to predict the prognosis results of patients [25,26]. In our research, we also predict the effect of immune therapy. They sometimes lack the exploration of the possible mechanism between the patients of various groups. In addition, our research found it may be related with the infiltration levels of immune cells. In addition, we also validate our prediction accuracy in five datasets which makes it more dependable. Besides, we explore the relationship between the risk scores and drug sensitivity. In addition, wet experiment is conducted which could be lacked in other studies. 

Based on machine learning methods, we constructed a prognostic signature to calculate the survival probability of BLCA and to further investigate the underlying mechanism of the ferroptosis-related signature. We found that the signature was not only correlated with the prognostic value but was also associated with the tumor microenvironment (TME), tumor mutation burden (TMB) and the curative outcomes of both immunotherapy and chemotherapy. Furthermore, we proved the reliability of the signature in some external datasets and built a risk score evaluation nomogram for clinical use. 

Rising evidence demonstrates ferroptosis plays a crucial role in tumor progression and metastasis, antitumor immunity, and drug resistance through ferroptosis regulators. Most studies have put stress on a single ferroptosis regulatory gene, but the mutual association and biological values of multiple ferroptosis regulators in tumor are not fully researched. For BLCA, even single gene studies related to ferroptosis are scarce. Here, we analyzed 113 ferroptosis regulators, including suppressors, drivers and markers, in BLCA. 

First, we depicted the genetic, mRNA and prognostic landscape of these ferroptosis regulators, including somatic mutation, copy number variation, expression level and hazard ratio of overall survival. Then, we built a scoring model according to the results of LASSO and multivariate Cox regression and a ferroptosis regulator signature to forecast the outcomes of individual patients. The model has been verified in five independent datasets and is associated with immune-associated pathways. The infiltration levels of immune cells were greatly higher in the group of lower risk score, demonstrating a better immunotherapy benefit. It is in accordance with TMB score results and the TIDE value. The results from the IMvigor210 cohort, which received immunotherapy treatment, further verified the immunotherapy prediction efficacy of the model. In addition, the risk score was associated with the estimated IC50 of multiple drugs in GDSC, indicating that the risk score might also predict the treatment efficacy of chemotherapy.

It is not uncommon to build a prognostic prediction signature on the basis of gene expression in cancer research, but most of these studies have then failed to further explore the possible mechanism of the signature and explain its validity from the aspect of biological function. Therefore, our survival prediction signature focused on the related pathway alterations in addition to the prediction efficacy for survival probability or therapeutic treatment. From our perspective, the reason why our signature could predict the poor survival of the high-risk group in five independent BLCA cohorts was attributed to activated MYC, E2F and G2/M checkpoint-related pathways. Multiple studies have reported that the expression levels of E2F are strictly monitored during the cell cycle via multiple layers to influence tumor progression [27,28]. The oncogene MYC contributes to the genesis, drug tolerance and metastasis of many human cancers [29,30], and the G2/M checkpoint controls the cell cycle fate to precisely regulate cell proliferation and division [31,32]. EMT associated with extracellular matrix components is also involved in tumor metastasis and drug resistance, accelerating the proliferation and metastasis of cancer cells [33,34]. Tumor subtypes with high degrees of progression might be sensitive to chemotherapy. From the results of the GDSC analysis, we suggest that cisplatin, docetaxel, doxorubicin, etoposide, vinblastine, etc., may be better for patients in the high-risk group. 

The group of lower risk scores owned a tremendously better survival time with activated P53 and interferon-γ response pathways and a higher infiltration of CD8 T and TFH cells, which were protective factors. It was reported M1 macrophages can release IL-12, IL-16, INF-γ and other proinflammatory cytokines, activating the inflammatory response and eliminating tumor cells [35]. These traits were enriched in the group of lower risk score, indicating patients in the group of lower risk score possibly are sensitive to immunotherapy. Actually, the risk score could not only predict the immunotherapy efficacy in terms of TIDE value but also reflect the real response of urologic carcinoma patients receiving the treatment of anti-PD-L1 agents, and this score was conversely related to immune cell infiltration. Unacceptable side effects and a failure to produce a durable response with the use of chemotherapeutic agents have led to immunotherapeutic agents joining the arsenal for the therapy of patients with BLCA. Our research added a novel method for forecasting the outcomes of immunotherapy.

From a deeper perspective, we found that IFNG was the most important member of the ferroptosis signature and was highly associated with immune-related pathways, antitumor immune cell infiltration and antigen presentation molecules. Previous studies have validated that IFNG could suppress SLC7A11, causing reduced cystine uptake, enhanced tumor lipid oxidation and ferroptosis, and improved tumor control [36]. However, the function of ferroptosis in drug resistance and immune evasion remains uncertain [37]. Our study identified a ferroptosis regulator signature that might take part in the process of antitumor immunity, indicating that these members might alter the ferroptosis level to regulate the immune process. For instance, prominin2 (PROM2), which could promote the shape of multivesicular bodies containing ferritin and exosomes delivering iron out of the cell so as to inhibit ferroptosis [38], was negatively associated with immune-associated pathways and the infiltration levels of antitumor immune cells. Through conducting correlation analysis of the expression of ferroptosis regulators and the index of ferroptosis, we further validated that most of them were highly associated with the ferroptosis process. Since some regulators exhibited some inconsistency with the FPI, this still needs to be validated by experimental studies in the future to ascertain the precise regulatory mechanisms.

While immunotherapy is widely reported to be a novel treatment for cancer, researches have demonstrated only about twenty percent of patients with solid tumor can gain positive effect from this type of therapy [39]. As a result, various studies have put stress on looking for biomarkers owing good ability of prediction in the treatment effect of immunotherapy. Many clinical indicators, such as the expression of PD-L1 [40], CD8+ T cells [41], TMB [23] and microsatellite instability (MSI) [42], are currently adopted for forecasting the effect and efficiency of immunotherapy treatment. While there were other elements or signatures on the basis of indicators related to the immune response, our research suggests that the expression pattern of ferroptosis regulators could also be useful in predicting the effect of immunotherapy.

## 5. Conclusions

Totally speaking, our systematic, integrated analysis of ferroptosis regulators suggests possible ways how the ferroptosis regulators impact the tumor microenvironment and their association with survival outcomes of the patients with BCa. A gene signature was established to identify their prediction effect both in chemotherapy and immunotherapy. This study puts stress on the critical clinical values of ferroptosis regulators and will help to develop personalized therapeutic strategies for BCa patients.

## 6. Limitation

Although we conducted the experiment to initially validate some results of bioinformatic analysis. There still needs some mechanism experiments to explore the reason that these genes could influence the prognosis results of patients with BCa. In addition, owing to that some researches that investigate the ferroptosis-related genes were not published yet at the time we finished our research, some associated genes were not included in the analysis.

## Data Availability

The datasets analyzed for this study are available from Gene expression Omnibus (https://www.ncbi.nlm.nih.gov/geo/, accessed on 2 February 2021), TCGA (https://portal.gdc.cancer.gov/, accessed on 2 February 2021), Xena database. The gene expression profiles and associated clinical parameters of 348 patients with metastatic urothelial cancer who accepted immune checkpoint inhibitor therapy were obtained from the database (http://researchpub.gene.com/IMvigor210CoreBiologies, accessed on 2 February 2021).

## Supplementary Materials

The following are available online at https://www.mdpi.com/article/10.3390/cancers13236069/s1, Table S1: The List of Ferroptosis Regulators Used in This Study, Table S2: The Immune Cell Signature Used in This Work for Estimating the Relative Abundance of Immune Cell Infiltration. Table S3: Primers Details of Genes Involved in Signature, Table S4: Parameters of Multivariate Cox Regression Model in TCGA-BLCA Cohort.
