# Supplementary material for: Identification of Survival and Therapeutic Response-Related Ferroptosis Regulators in Bladder Cancer through Data Mining and Experimental Validation
Source: Cancers (Basel). 2021 Dec 2;13(23):6069. doi: 10.3390/cancers13236069 (PMC8656535; doi:10.3390/cancers13236069)
Supplement: Supplementary file 1 [file cancers-13-06069-s001.zip › cancers-1457240-supplementary.pdf]

Table S1. The list of ferroptosis regulators uesd in this study.

Symbol	Confidence	Type
ACSL3	Validated	suppressor
AIFM2	Validated	suppressor
AKR1C1	Validated	suppressor
AKR1C2	Validated	suppressor
AKR1C3	Validated	suppressor
ARNTL	Validated	suppressor
ATF4	Validated	suppressor
BRD4	Validated	suppressor
CA9	Validated	suppressor
CAV1	Validated	suppressor
CBS	Validated	suppressor
CD44	Validated	suppressor
CDKN1A	Validated	suppressor
CHMP5	Validated	suppressor
CHMP6	Validated	suppressor
CISD1	Validated	suppressor
CISD2	Validated	suppressor
FADS2	Validated	suppressor
FH	Validated	suppressor
GCH1	Validated	suppressor
GPX4	Validated	suppressor
HELLS	Validated	suppressor
HIF1A	Validated	suppressor
HSF1	Validated	suppressor
HSPA5	Validated	suppressor
HSPB1	Validated	suppressor
ISCU	Validated	suppressor
LAMP2	Validated	suppressor
MT1G	Validated	suppressor
MUC1	Validated	suppressor
NF2	Validated	suppressor
NFE2L2	Validated	suppressor
NFS1	Validated	suppressor
NQO1	Validated	suppressor
OTUB1	Validated	suppressor
PML	Validated	suppressor
PRDX6	Validated	suppressor
PROM2	Validated	suppressor
RB1	Validated	suppressor
SCD	Validated	suppressor
SESN2	Validated	suppressor
SLC40A1	Validated	suppressor
SLC7A11	Validated	suppressor
SQSTM1	Validated	suppressor
SRC	Validated	suppressor
STAT3	Validated	suppressor
TMBIM4	Validated	suppressor
TP63	Validated	suppressor
ZFP36	Validated	suppressor
CHAC1	Validated	marker
FTH1	Validated	marker
PTGS2	Validated	marker
ABCC1	Validated	driver
ACSF2	Validated	driver
ACSL4	Validated	driver
ACVR1B	Validated	driver
ALOX12	Validated	driver

ALOX12B	Validated	driver
ALOX15	Validated	driver
ALOX15B	Validated	driver
ALOX5	Validated	driver
ALOXE3	Validated	driver
ANO6	Validated	driver
ATF3	Validated	driver
ATG5	Validated	driver
ATG7	Validated	driver
ATM	Validated	driver
ATP5G3	Validated	driver
BAP1	Validated	driver
BECN1	Validated	driver
CARS	Validated	driver
CDKN2A	Validated	driver
CDO1	Validated	driver
CS	Validated	driver
DPP4	Validated	driver
EGFR	Validated	driver
EGLN2	Validated	driver
ELAVL1	Validated	driver
EMC2	Validated	driver
G6PD	Validated	driver
GOT1	Validated	driver
HMGB1	Validated	driver
HMOX1	Validated	driver
IDH1	Validated	driver
IFNG	Validated	driver
IREB2	Validated	driver
KEAP1	Validated	driver
LONP1	Validated	driver
MAPK1	Validated	driver
MAPK3	Validated	driver
MIOX	Validated	driver
MTDH	Validated	driver
MYB	Validated	driver
NCOA4	Validated	driver
NOX4	Validated	driver
PANX1	Validated	driver
PEBP1	Validated	driver
PGD	Validated	driver
PHKG2	Validated	driver
PRKAA1	Validated	driver
PRKAA2	Validated	driver
RPL8	Validated	driver
SAT1	Validated	driver
SLC1A5	Validated	driver
SOCS1	Validated	driver
TAZ	Validated	driver
TF	Validated	driver
TGFBR1	Validated	driver
TNFAIP3	Validated	driver
TP53	Validated	driver
VDAC2	Validated	driver
YY1AP1	Validated	driver
ZEB1	Validated	driver

Table S2. The immune cell signature used in this work for estimating the relative abundance of immune cell infiltration.

CellType	Gene_Symbol
aDC	LAMP3
aDC	CCL1
aDC	IDO1
aDC	OAS3
aDC	EBI3
B cells	GLDC
B cells	COCH
B cells	SLC15A2
B cells	CR2
B cells	HLA-DOB
B cells	SPIB
B cells	PNOC
B cells	BLK
B cells	CD19
B cells	KIAA0125
B cells	TNFRSF17
B cells	GNG7
B cells	CCR9
B cells	BLNK
B cells	ABCB4
B cells	MEF2C
B cells	MEF2C
B cells	IGHM
B cells	TCL1A
B cells	BCL11A
B cells	MS4A1
B cells	SCN3A
B cells	MICAL3
B cells	IGHD
B cells	IGHA1
B cells	IGKC
B cells	DTNB
B cells	CD72
B cells	IGLC1
B cells	MS4A1
B cells	QRSL1
B cells	QRSL1
B cells	OSBPL10
B cells	BCL11A
B cells	KIAA0125
B cells	BACH2
B cells	FCRL2
B cells	IGHD
B cells	TCL1A
CD8 T cells	DNAJB1
CD8 T cells	DNAJB1
CD8 T cells	ZFP36L2
CD8 T cells	ZFP36L2
CD8 T cells	VAMP2
CD8 T cells	PPP1R2
CD8 T cells	TBCC
CD8 T cells	LEPROTL1
CD8 T cells	CAMLG
CD8 T cells	KLF9
CD8 T cells	GADD45A
CD8 T cells	CD8A
CD8 T cells	ZNF91
CD8 T cells	PF4

CD8 T cells	THUMPD1
CD8 T cells	TSC22D3
CD8 T cells	SLC16A7
CD8 T cells	GZMM
CD8 T cells	SIK1
CD8 T cells	RBM3
CD8 T cells	APBA2
CD8 T cells	HAUS3
CD8 T cells	SF1
CD8 T cells	FLT3LG
CD8 T cells	TMEM259
CD8 T cells	ZNF609
CD8 T cells	SRSF7
CD8 T cells	PRF1
CD8 T cells	TMC6
CD8 T cells	KAT6A
CD8 T cells	AES
CD8 T cells	ZNF22
CD8 T cells	ABT1
CD8 T cells	CDKN2AIP
CD8 T cells	PRR5
CD8 T cells	LIME1
CD8 T cells	PRR5
CD8 T cells	C12orf47
Cytotoxic cells	RUNX3
Cytotoxic cells	DUSP2
Cytotoxic cells	GZMA
Cytotoxic cells	GNLY
Cytotoxic cells	ZBTB16
Cytotoxic cells	KLRD1
Cytotoxic cells	KLRD1
Cytotoxic cells	APBA2
Cytotoxic cells	GZMH
Cytotoxic cells	RORA
Cytotoxic cells	KLRD1
Cytotoxic cells	NKG7
Cytotoxic cells	CTSW
Cytotoxic cells	KLRB1
Cytotoxic cells	SIGIRR
Cytotoxic cells	KLRF1
Cytotoxic cells	APOL3
Cytotoxic cells	GNLY
Cytotoxic cells	SIGIRR
DC	HSD11B1
DC	CCL13
DC	CD209
DC	CCL22
DC	CCL17
DC	PPFIBP2
DC	NPR1
Eosinophils	THBS1
Eosinophils	THBS1
Eosinophils	SIAH1
Eosinophils	TGIF1
Eosinophils	HES1
Eosinophils	HES1
Eosinophils	KBTBD11
Eosinophils	THBS4
Eosinophils	KCNH2
Eosinophils	ABHD2
Eosinophils	RNASE2
Eosinophils	CLC

Eosinophils	PTGDR2
Eosinophils	EMR1
Eosinophils	CD101
Eosinophils	SYNJ1
Eosinophils	CCR3
Eosinophils	HIST1H1C
Eosinophils	KCNH2
Eosinophils	IL5RA
Eosinophils	IL5RA
Eosinophils	GALC
Eosinophils	CAT
Eosinophils	TIPARP
Eosinophils	SYNJ1
Eosinophils	TKTL1
Eosinophils	LRP5L
Eosinophils	RRP12
Eosinophils	TKTL1
Eosinophils	RCOR3
Eosinophils	SMPD3
Eosinophils	CYSLTR2
Eosinophils	HRH4
Eosinophils	ACACB
Eosinophils	C9orf156
Eosinophils	ACACB
Eosinophils	ACACB
Eosinophils	MYO15B
iDC	BLVRB
iDC	TACSTD2
iDC	CSF1R
iDC	F13A1
iDC	GSTT1
iDC	RAP1GAP
iDC	VASH1
iDC	FABP4
iDC	PREP
iDC	MMP12
iDC	CTNS
iDC	SYT17
iDC	CD1C
iDC	GUCA1A
iDC	CLEC10A
iDC	CD1B
iDC	CH25H
iDC	PPARG
iDC	CD1E
iDC	ABCG2
iDC	FZD2
iDC	CD1A
iDC	CD1E
iDC	SLC7A8
iDC	PDXK
iDC	NUDT9
iDC	MS4A6A
iDC	HS3ST2
iDC	DCSTAMP
iDC	LMAN2L
Macrophages	EMP1
Macrophages	SCARB2
Macrophages	RAI14
Macrophages	CTSK
Macrophages	APOE
Macrophages	CD163

Macrophages	FDX1
Macrophages	SCG5
Macrophages	CYBB
Macrophages	ME1
Macrophages	GPC4
Macrophages	GPC4
Macrophages	KAL1
Macrophages	SULT1C2
Macrophages	MARCO
Macrophages	CCL7
Macrophages	CHIT1
Macrophages	MSR1
Macrophages	CHI3L1
Macrophages	CD84
Macrophages	SULT1C2
Macrophages	PTGDS
Macrophages	MSR1
Macrophages	PTGDS
Macrophages	SGMS1
Macrophages	BCAT1
Macrophages	BCAT1
Macrophages	FN1
Macrophages	MSR1
Macrophages	CXCL5
Macrophages	CD163
Macrophages	GM2A
Macrophages	ATG7
Macrophages	PCOLCE2
Macrophages	MS4A4A
Macrophages	CLEC5A
Macrophages	DNASE2B
Macrophages	COLEC12
Macrophages	COL8A2
Mast cells	ABCC4
Mast cells	MPO
Mast cells	SCG2
Mast cells	MAOB
Mast cells	VWA5A
Mast cells	KIT
Mast cells	PTGS1
Mast cells	CALB2
Mast cells	CPA3
Mast cells	CTSG
Mast cells	TPSAB1
Mast cells	SLC18A2
Mast cells	ADCYAP1
Mast cells	TAL1
Mast cells	SIGLEC6
Mast cells	SIGLEC6
Mast cells	NR0B1
Mast cells	CEACAM8
Mast cells	HPGDS
Mast cells	ELANE
Mast cells	HDC
Mast cells	TPSB2
Mast cells	MS4A2
Mast cells	MS4A2
Mast cells	TPSAB1
Mast cells	GATA2
Mast cells	TPSAB1
Mast cells	VWA5A
Mast cells	SIGLEC6

Mast cells	HPGD
Mast cells	PRG2
Mast cells	PPM1H
Mast cells	CMA1
Mast cells	LINC01140
Mast cells	TPSAB1
Mast cells	MLPH
Mast cells	SLC24A3
Mast cells	SLC24A3
Neutrophils	CD93
Neutrophils	CD93
Neutrophils	MME
Neutrophils	MME
Neutrophils	CSF3R
Neutrophils	FCGR3B
Neutrophils	TECPR2
Neutrophils	FPR1
Neutrophils	BST1
Neutrophils	S100A12
Neutrophils	SLC22A4
Neutrophils	TNFRSF10C
Neutrophils	CYP4F3
Neutrophils	MGAM
Neutrophils	CXCR2
Neutrophils	CXCR1
Neutrophils	FCAR
Neutrophils	CEACAM3
Neutrophils	LILRB2
Neutrophils	FPR2
Neutrophils	FPR2
Neutrophils	CEACAM3
Neutrophils	TNFRSF10C
Neutrophils	FCAR
Neutrophils	KCNJ15
Neutrophils	FCAR
Neutrophils	G0S2
Neutrophils	ALPL
Neutrophils	SLC25A37
Neutrophils	CPPED1
Neutrophils	DYSF
Neutrophils	HPSE
Neutrophils	SIGLEC5
Neutrophils	VNN3
Neutrophils	CRISPLD2
NK CD56bright cells	DUSP4
NK CD56bright cells	DUSP4
NK CD56bright cells	PLA2G6
NK CD56bright cells	RRAD
NK CD56bright cells	FOXJ1
NK CD56bright cells	XCL1
NK CD56bright cells	MPPED1
NK CD56bright cells	PLA2G6
NK CD56bright cells	MUC3B
NK CD56bright cells	LPCAT4
NK CD56bright cells	PLA2G6
NK CD56bright cells	TRAPPC9
NK CD56bright cells	MADD
NK CD56dim cells	GTF3C1
NK CD56dim cells	KIR2DL3
NK CD56dim cells	KIR2DS5
NK CD56dim cells	GZMB
NK CD56dim cells	KIR3DS1

NK CD56dim cells	KIR2DS2
NK CD56dim cells	KIR3DL1
NK CD56dim cells	TTC38
NK CD56dim cells	KIR2DS4
NK CD56dim cells	KIR3DL3
NK CD56dim cells	PMEPA1
NK CD56dim cells	S1PR5
NK CD56dim cells	IL21R
NK cells	IGFBP5
NK cells	MAPRE3
NK cells	ZNF747
NK cells	XCL1
NK cells	ZNF205
NK cells	TCTN2
NK cells	TRPV6
NK cells	FGF18
NK cells	BCL2
NK cells	TBXA2R
NK cells	NCR1
NK cells	CDC5L
NK cells	MRC2
NK cells	FZR1
NK cells	ALDH1B1
NK cells	ADARB1
NK cells	PSMD4
NK cells	FUT5
NK cells	FZR1
NK cells	APBB2
NK cells	PDLIM4
NK cells	ZNF528
NK cells	MCM3AP
NK cells	LDB3
NK cells	GNAS
NK cells	NCR1
NK cells	NCR1
NK cells	TINAGL1
NK cells	PRX
NK cells	SLC30A5
NK cells	KANK2
pDC	IL3RA
T cells	ITM2A
T cells	LCK
T cells	LCK
T cells	CD3E
T cells	SKAP1
T cells	CD2
T cells	CD28
T cells	CD96
T cells	CD3G
T cells	TRAC
T cells	TRAC
T cells	PRKCQ
T cells	PRKCQ
T cells	SH2D1A
T cells	TRBC1
T cells	NCALD
T cells	TRBC1
T cells	CD3D
T cells	YME1L1
T cells	CD6
T cells	TRDV3
T cells	TRAT1

T cells	BCL11B
T helper cells	UBE2L3
T helper cells	ANP32B
T helper cells	NAP1L4
T helper cells	RPA1
T helper cells	SEC24C
T helper cells	ITM2A
T helper cells	SLC25A12
T helper cells	ASF1A
T helper cells	ATF2
T helper cells	BATF
T helper cells	GOLGA8A
T helper cells	ICOS
T helper cells	CD28
T helper cells	FRYL
T helper cells	PPP2R5C
T helper cells	SRSF10
T helper cells	LRBA
T helper cells	FAM111A
T helper cells	NUP107
T helper cells	PHF10
T helper cells	BORA
T helper cells	DDX50
Tcm	SLC7A6
Tcm	AQP3
Tcm	CLUAP1
Tcm	FYB
Tcm	USP9Y
Tcm	TXK
Tcm	KLF12
Tcm	CASP8
Tcm	CEP68
Tcm	ATM
Tcm	PCM1
Tcm	CDC14A
Tcm	NFATC3
Tcm	TIMM8A
Tcm	ATM
Tcm	HNRNPH1
Tcm	CREBZF
Tcm	TXLNGY
Tcm	CYLD
Tcm	MAP3K1
Tcm	DOCK9
Tcm	N4BP2L2-IT2
Tcm	PCNX
Tcm	REPS1
Tcm	TRAF3IP3
Tcm	PHC3
Tcm	NMT2
Tcm	KMT2A
Tcm	SPDYE2
Tcm	STX16
Tcm	NEFL
Tem	TBCD
Tem	TBC1D5
Tem	NFATC4
Tem	PRKY
Tem	CCR2
Tem	LTK
Tem	CCR2
Tem	MEFV

Tem	DDX17
Tem	SND1-IT1
Tem	FLI1
Tem	FLI1
Tem	AKT3
Tem	EZR
Tem	TCRA
Tem	GDPD5
TFH	STK39
TFH	PTPN13
TFH	CHGB
TFH	TOX
TFH	CDK5R1
TFH	CXCL13
TFH	PVALB
TFH	CXCR5
TFH	MAF
TFH	PDCD1
TFH	ICA1
TFH	LDLRAD4
TFH	MYO7A
TFH	MAF
TFH	LDLRAD4
TFH	TSHR
TFH	ST8SIA1
TFH	MYO6
TFH	ICA1
TFH	SMAD1
TFH	CHI3L2
TFH	PASK
TFH	HIST1H4J
TFH	CXCR5
TFH	PASK
TFH	MKL2
TFH	POMT1
TFH	MAGEH1
TFH	HEY1
TFH	SH3TC1
TFH	B3GAT1
TFH	KCNK5
TFH	THADA
TFH	SIRPG
TFH	SLC7A10
TFH	KIAA1324
TFH	MYO7A
TFH	HEY1
TFH	THADA
TFH	ZNF764
Tgd	FEZ1
Tgd	C1orf61
Tgd	CD160
Tgd	TRDC
Th1 cells	APOD
Th1 cells	BST2
Th1 cells	DPP4
Th1 cells	CCL4
Th1 cells	SGCB
Th1 cells	LRP8
Th1 cells	BTG3
Th1 cells	SYNGR3
Th1 cells	CD38
Th1 cells	CD70

Th1 cells	DGKI
Th1 cells	LTA
Th1 cells	IL12RB2
Th1 cells	DUSP5
Th1 cells	LRRN3
Th1 cells	CSF2
Th1 cells	IFNG
Th1 cells	CMAHP
Th1 cells	DPP4
Th1 cells	ATP9A
Th1 cells	APBB2
Th1 cells	DOK5
Th1 cells	EGFL6
Th1 cells	ZBTB32
Th1 cells	IL22
Th1 cells	CTLA4
Th1 cells	HBEGF
Th1 cells	APBB2
Th17 cells	IL17RA
Th17 cells	RORC
Th17 cells	IL17A
Th17 cells	IL17A
Th2 cells	DHFR
Th2 cells	SNRPD1
Th2 cells	GSTA4
Th2 cells	SMAD2
Th2 cells	SMAD2
Th2 cells	MB
Th2 cells	CDC7
Th2 cells	WDHD1
Th2 cells	CDC25C
Th2 cells	PMCH
Th2 cells	CXCR6
Th2 cells	LAIR2
Th2 cells	PTGIS
Th2 cells	ANK1
Th2 cells	CENPF
Th2 cells	GATA3
Th2 cells	EVI5
Th2 cells	BIRC5
Th2 cells	PHEX
Th2 cells	CXCR6
Th2 cells	SLC39A14
Th2 cells	MICAL2
Th2 cells	ADCY1
Th2 cells	LIMA1
Th2 cells	NEIL3
Th2 cells	HELLS
Th2 cells	AHI1
Th2 cells	AHI1
Th2 cells	IL26
Th2 cells	AHI1
Th2 cells	DHFR
TReg	FOXP3
TReg	FOXP3

Table S3. Primers details of genes involved in signature.

Gene	Forward	Reverse
G6PD	AGAGCTTTTCCAGGGCGATG	ACGATGAAGGTGTTTTCGGGC
EGFR	ATCCCACAGCAGGGCTTCTTCA	AGCAGGACTGTTTCCAGACAAGC
CHMP6	ACAGGACGGAGAACCAGATCA	CTCCACCTCTTCAATGGACATCA
AIM2	TTCTCTGCACCGGCATCAAGA	AATGGCGTAGACGTTGCTGTG
VDAC2	AACTTTGCAGTGGGCTACAGG	TGCCAAAACGAGTGCAGTTGG
IFNG	CAGCTCTGCATCGTTTTGGGT	CCGCTACATCTGAATGACCTGC
PROM2	TCTCGGTGGTGCAGCTCAAT	TGTGCTCTGTCTTCACTCGTCC

Table S4. Parameters of multivariate Cox regression model in TCGA-BLCA cohort.

id	coef	exp(coef)	se(coef)	z	Pr(>|z|)
CHMP6	0.35358744	1.424167507	0.13640893	2.59211364	0.009538826
VDAC2	0.35189238	1.421755507	0.12672778	2.7767581	0.005490402
G6PD	0.21776994	1.243301	0.07128877	3.05475818	0.002252421
PROM2	-0.167244	0.845993158	0.03871818	-4.3195218	0.000015637
EGFR	0.17468025	1.190865374	0.04817747	3.62576645	0.000288106
IFNG	-0.3193266	0.726638202	0.08028624	-3.9773515	0.000069687
AIFM2	-0.3695087	0.691073755	0.10013156	-3.6902323	0.000224049
